# Down-Regulation of Flagellar, Fimbriae, and Pili Proteins in Carbapenem-Resistant *Klebsiella pneumoniae* (NDM-4) Clinical Isolates: A Novel Linkage to Drug Resistance

**DOI:** 10.3389/fmicb.2019.02865

**Published:** 2019-12-17

**Authors:** Divakar Sharma, Anjali Garg, Manish Kumar, Faraz Rashid, Asad U. Khan

**Affiliations:** ^1^Interdisciplinary Biotechnology Unit, Aligarh Muslim University, Aligarh, India; ^2^Department of Biophysics, University of Delhi, New Delhi, India; ^3^SCIEX Pvt. Ltd., Gurgaon, India

**Keywords:** *Klebsiella pneumoniae* (NDM-4), proteomics, bioinformatics, pathway enrichment, biofilm, carbapenem resistance

## Abstract

The emergence and spread of carbapenem-resistant *Klebsiella pneumoniae* infections have worsened the current situation worldwide, in which totally drug-resistant strains (bad bugs) are becoming increasingly prominent. Bacterial biofilms enable bacteria to tolerate higher doses of antibiotics and other stresses, which may lead to the drug resistance. In the present study, we performed proteomics on the carbapenem-resistant NDM-4-producing *K. pneumonia*e clinical isolate under meropenem stress. Liquid chromatography coupled with mass spectrometry (LC–MS/MS) analysis revealed that 69 proteins were down-regulated (≤0.42-fold change) under meropenem exposure. Within the identified down-regulated proteome (69 proteins), we found a group of 13 proteins involved in flagellar, fimbriae, and pili formation and their related functions. Further, systems biology approaches were employed to reveal their networking pathways. We suggest that these down-regulated proteins and their interactive partners cumulatively contribute to the emergence of a biofilm-like state and the survival of bacteria under drug pressure, which could reveal novel mechanisms or pathways involved in drug resistance. These down-regulated proteins and their pathways might be used as targets for the development of novel therapeutics against antimicrobial-resistant (AMR) infections.

## Introduction

*Klebsiella pneumoniae* is a gram-negative bacteria of the family Enterobacteriaceae. In clinical settings, the emergence and spread of drug-resistant *K. pneumoniae* are worsening the medical situation worldwide. Carbapenems have been considered the last line of defense in the treatment of drug-resistant infections (Paterson, 2000; Paterson and Bonomo, 2005). Interrupted use of carbapenem during the course of treatment leads to the emergence of carbapenem-resistant infections. Carbapenemases are produced that cleave or hydrolyze the carbapenem drugs and contribute to carbapenem resistance. Carbapenemase over-production and porin deficiency are the two major causes of carbapenem resistance (Ambler et al., 1991; Martínez-Martínez et al., 1999; Jacoby et al., 2004; Loli et al., 2006). Several explanations have been put forward to explain the mechanisms of carbapenem resistance, but our information is as yet incomplete or fragmentary.

Biofilm formation is among the mechanisms known to be responsible for microbial drug resistance. During biofilm formation, bacteria first become sessile and then colonize and grow up from surfaces. The biofilm protects the bacteria from various stresses like altered pH, osmolarity, and nutrient scarcity (Costerton and Lewandowski, 1995; Fux et al., 2005; McCarty et al., 2012) and blocks the entry of drugs to the bacterial communities (Costerton et al., 1999; Stewart and William Costerton, 2001; Sharma et al., 2019c). In the first step of biofilm formation, bacteria lose their motility and become sessile. We assume that decreased expression of proteins related to motility could lead to biofilm formation and thus might contribute to the development of drug resistance. Comparative proteomics addressing the whole-cell proteins of drug-resistant microbes with or without drug pressures have been reported previously (Lata et al., 2015; Khan et al., 2017; Sharma et al., 2018a; Qayyum et al., 2019; Sharma et al., 2019a). However, little information is available regarding the bacterial proteome related to biofilm, and, to the best of our knowledge, no data has yet been reported related to the proteome of drug-resistant microbes, especially carbapenem-resistant *K. pneumonia*, in relation to motility-mediated drug resistance.

In this study, we used comparative proteomics and systems biology-based approaches to investigate the correlation of the decreased expression of motility-related proteins (flagellar, fimbriae, and pili) with biofilm formation, which may lead to the development of drug resistance. Proteomics and systems biology approaches are both among the potential strategies for exploring biological problems such as the mechanisms of drug resistance. In the present study, we used liquid chromatography coupled with mass spectrometry (LC–MS/MS) to determine the expression of the motility-related proteome of a carbapenem-resistant *K. pneumoniae* (NDM-4) clinical isolate under meropenem stress. The results of this study could lead to the exploration of novel therapeutics targets against carbapenem resistance.

## Materials and Methods

### Strain Selection and Drug Susceptibility Testing

An NDM-4-encoding carbapenem-resistant *K. pneumoniae* clinical isolate (AK-97) was selected for this study. This was reported in our earlier study, which showed its presence in the NICU of a northern Indian Hospital (Ahmad et al., 2018). Drug susceptibility testing (DST) against the drug meropenem was carried out via the micro-dilution method according to CLSI guidelines (Wayne, 2014).

### Culture Scaling, Drug Induction, and Protein Sample Preparation

A single colony of *K. pneumoniae* was inoculated in LB broth and kept at 37°C at 220 rpm, and a sub-MIC (32 μg/ml) of meropenem was used for induction in a 200 ml culture flask. Bacteria were grown up to the exponential phase (OD_600_ = 0.8), and cells were harvested by centrifugation at 8000 × *g* for 8 min at 4°C. The cells were washed with normal saline and re-suspended in a lysis buffer [50 mM Tris–HCl containing 10 mM MgCl_2_, 0.1% sodium azide, 1 mM phenyl-methyl-sulfonyl-fluoride (PMSF), and 1 mM ethylene glycol tetra-acetic acid (EGTA); pH 7.4] at a concentration of 1 g wet weight per 5 ml. Cell lysis was performed by intermittent sonication with a sonicator with the power at 35% amplitude (Sonics & Materials Inc., Newtown, CT, United States) for 10 min at 4°C. Further, the homogenate was centrifuged at 12,000 × *g* for 20 min at 4°C, and the supernatant was precipitated overnight at −20°C by adding cold acetone in excess (1:4) (Lata et al., 2015; Sharma and Bisht, 2016; Sharma et al., 2019a). The precipitated protein was collected by centrifugation (12,000 × *g*, 20 min), allowed to air dry, and then suspended in an appropriate volume of protein-dissolving buffer. The protein concentration was estimated using the Bradford (1976) assay. All of the experiments were replicated biologically and technically.

### Separation and Identification of the Proteome by nanoLC-TripleTOF 5600 MS

Equal concentrations of protein samples were trypsinized, and digested proteins were analyzed using a TripleTOF 5600 MS (AB Sciex, Foster City, CA, United States) equipped with an Eksigent MicroLC 200 system (Eksigent, Dublin, CA, United States) with an Eksigent C18 reverse-phase column (150 × 0.3 mm, 3 μm, 120 Å) (Sharma et al., 2019a). For protein identification, spectral libraries were generated using information-dependent acquisition (IDA) mode after injecting 2 gm of tryptic digest on the column using an Eksigent NanoLC-Ultra^TM^ 2D Plus system coupled with a SCIEX Triple TOF^®^ 5600 system fitted with a NanoSpray III source. The samples were loaded on the trap (Eksigent Chrom XP 350 μm × 0.5 mm, 3 μm, 120 Å) and washed for 30 min at 3 μl/min. A 120 min gradient in multiple steps (ranging from 5 to 50% acetonitrile in water containing 0.1% formic acid) was set up to elute the peptides from the ChromXP 3-C18 (0.075 × 150 mm, 3 μm, 120 Å) analytical column. Technical replicates of the nanoLC-TripleTOF 5600 MS experiments were performed.

### Sequential Window Acquisition of all Theoretical Fragment Ion Spectra (SWATH) Analysis for Label-Free Quantification

For label-free quantification (SWATH analysis), data-dependent analysis (DDA) mode was applied for both samples to generate high-quality spectral ion libraries by operating the mass spectrometer with specific parameters (Sharma and Bisht, 2016). In the SWATH acquisition method, the Q1 transmission window was set to 12 Da from the mass range 350–1250 Da. A total of 75 windows were acquired independently with an accumulation time of 62 ms, along with three technical replicates for each of the sets. The total cycle time was kept constant at <5 s. Protein Pilot^TM^ v. 5.0 was used to generate the spectral library. For label-free quantification, peak extraction and spectral alignment were performed using PeakView^®^ 2.2 Software with the parameters set as follows: number of peptides, 2; number of transitions, 5; peptide confidence, 95%; XIC width, 30 ppm; XIC extraction window, 3 min. The data were further processed in MarkerView software v. 1.3 (AB Sciex, Foster City, CA, United States) for statistical data interpretation. In MarkerView, the peak area under the curve (AUC) for the selected peptides was normalized by the internal standard protein (beta-galactosidase) spike during SWATH accumulation. The results were extracted as three output files containing the AUC of the ions, the summed intensity of peptides for protein, and the summed intensity of ions for the peptide. All SWATH acquisition data were processed using SWATH Acquisition MicroApp 2.0 in PeakView^®^ Software.

### Data Analysis

Data were processed with Protein Pilot Software v. 5.0 (AB Sciex, Foster City, CA, United States) utilizing the Paragon and Progroup Algorithm. The analysis was done using the tools integrated into Protein Pilot at a 1% false discovery rate (FDR) with statistical significance. In brief, the UniProt database searched for the *K. pneumoniae* taxonomy, which was download from the database in July 2018. The download included total combined (reviewed and un-reviewed) entries of 409,060 proteins. We used cRAP analysis to identify the proteins commonly found in proteomics experiments (unavoidable contamination) of protein samples. *E. coli* beta-galactosidase (BGAL_ECOLI-[P00722]) was used as a molecular weight marker to calibrate the system for sample acquisition. The internal standard was used for the normalization of statistical parameters. We exported the label-free quantified data and imported them into MarkerView software V1.3 to obtain statistical data for further interpretation. Triplicate data for each sample were normalized using the internal protein (beta-galactosidase) area, which was initially spiked in the samples. After normalization, principal component analysis (PCA) was performed to check the possible correlated variables within the group. We plotted a volcano curve to determine the statistical significant fold change versus *p*-value for the control and test. Proteins with a significant fold change < 0.42 were considered down-regulated proteins. Peak extraction and spectral alignment were performed using PeakView software (v. 2.2, AB Sciex, Foster City, CA, United States) with the following parameter settings: number of peptides per protein, 5; number of transitions per peptide, 6; selected peptide confidence, 1% FDR; XIC width, 30 ppm; XIC extraction window, 3 min.

### Gene Ontology Term Assignment and Analysis

*Klebsiella pneumonia* subsp. *pneumoniae* (strain ATCC 700721/MGH 78578) was used as a reference strain to carry out the functional studies. The proteins obtained from LC–MS/MS were firstly aligned to the reference strain proteome. Reference strain proteins that showed alignment identity of ≥50% over 80% of the sequence length of the reference strain protein were considered as homologs of the LC–MS/MS identified proteins. The Gene Ontology (GO) terms associated with the reference strain protein were used for the functional annotation. We used the slim version of GO terms, which were obtained from the Gene Ontology Consortium^1^ (Camon et al., 2003).

### Protein–Protein Interaction Network Integration

To find the interaction partner(s) of down-regulated proteins, protein–protein interaction (PPI) information were obtained from the STRING database v10.0^2^ and Cytoscape (version 3.6.1) (Shannon et al., 2003; Sharma and Bisht, 2017a,b,c; Sharma et al., 2018b, 2019b; Sharma and Khan, 2018). The PPI information provided by the STRING database has been established by experimental studies or by genomic analyses like domain fusion, phylogenetic profiling high-throughput experiments, co-expression studies, and gene neighborhood analysis. In the present study, only interactions with a score of ≥0.4 were used.

## Results

### Identification of Proteins by LC–MS/MS

In this study, we grew the carbapenem-resistant isolate at meropenem sub-MIC 32 mg/L. Further, we identified the down-regulated proteome of the same bacteria by LC–MS/MS using a SWATH workflow; 1156 proteins were quantified at 1% FDR with statistical significance as per the log fold change vs. *p*-value. Among them, 69 proteins were down-regulated (<0.42 log fold change vs. *p*-value) and are tabulated in the Supplementary Material (Supplementary Table S1).

In the present work, our main focus was on motility-related proteins. After critical analysis of the 69 down-regulated proteins, we found 13 proteins (around 19%) that belonged to the flagella-, fimbriae-, and pili-related protein functional groups (Table 1). These proteins are flagellar motor switch protein FliG, flagellar hook protein FlgE, negative regulator of flagellin synthesis FlgM, putative fimbriae major subunit StbA, flagellar hook-associated protein 2, chaperone FimC protein, flagellar biosynthesis protein FlgN, flagellar basal body protein, conjugal transfer protein TraC, fimbrial subunit type 1, chemotaxis regulator-transmits chemoreceptor signals to flagellar motor components CheY, and flagellin, all of which are involved in motility and its supporting processes.

**TABLE 1:** Details of the down-regulated proteome (flagella-, fimbriae-, and pili-related proteins) under meropenem stress in *Klebsiella pneumonia* clinical isolates (NDM-4).

**S. No.**	**Protein name**	**Log fold change vs. *p*-value**	**Accession number**	**Protein symbol**	**Matched organism strain**
1	Flagellar motor switch protein FliG	0.42	W1AYD1	FliG	*K. Pneumoniae* IS22
2	Flagellar hook protein FlgE	0.32	W1AUQ1	FlgE	*K. Pneumoniae* IS22
3	Negative regulator of flagellin synthesis FlgM	0.23	W1AWJ3	FlgM	*K. Pneumoniae* IS22
4	Putative fimbriae major subunit StbA	0.23	A6T548	StbA	*K. pneumoniae* subsp. *pneumoniae* (strain ATCC 700721/MGH 78578)
5	Flagellar hook-associated protein 2	0.22	W1B018	…….	*K. Pneumoniae* IS22
6	FimC protein	0.17	W1AP20	FimC	*K. Pneumoniae* IS22
7	Chaperone FimC	0.15	W1BBF2	FimC	*K. Pneumoniae* IS22
8	Flagellar biosynthesis protein FlgN	0.09	W1ATC5	FlgN	*K. Pneumoniae* IS22
9	Flagellar basal body protein	0.08	W1AT19	…….	*K. Pneumoniae* IS22
10	Conjugal transfer protein TraC	0.06	W1B0W2	TraC	*K. Pneumoniae* IS22
11	Fimbrial subunit type 1	0.05	W1B9X4	FimA	*K. Pneumoniae* IS22
12	Chemotaxis regulator-transmits chemoreceptor signals to flagelllar motor components CheY	0.04	W1BDF3	CheY	*K. Pneumoniae* IS22
13	Flagellin	0.01	W1AZS9	…….	*K. Pneumoniae* IS22

On the basis of the parameters described in the section “Materials and Methods,” we were able to map 67 out of the 69 down-regulated proteins on the proteome of the ATCC 700721/MGH 78578 strain of *K. pneumoniae* (Supplementary Table S2). Our GO results for down-regulated genes also show that most down-regulated proteins were involved in nitrogen and other small molecule metabolism, ion binding, and oxidoreductase activity (Table 2).

**TABLE 2:** Functional analysis of down-regulated genes associated with *Klebsiella pneumonia* subsp. *pneumonia* (strain ATCC 700721/MGH 78578).

**GO ID**	**Function**	**Gene name**	**No. of genes in which**
			**GO term was found**
**(A) Biological functions**			
GO:0005975	Carbohydrate metabolic process	*deoC*, *dhaK*, *dhaL*, *glk*, *gmhB*, *gnd*, *lacZ2*, *malP*, *talB*, *treC*, *uxaC*	11
GO:0006091	Generation of precursor metabolites and energy	*aspA*, *fdhF*, *glk*, *gor*	4
GO:0006259	DNA metabolic process	*ung*, *uvrD*	2
GO:0006399	tRNA metabolic process	*gltX*, *pheS*, *thrS*	3
GO:0006412	Translation	*gltX*, *pheS*, *thrS*	3
GO:0006457	Protein folding	*fimC*	1
GO:0006461	Protein complex assembly	*hscB*	1
GO:0006464	Cellular protein modification process	*ppiD*, *ptsH*	2
GO:0006520	Cellular amino acid metabolic process	*aspA*, *gcvH*, *gcvP*, *gcvT*, *gltX*, *hisD*, *ilvC*, *pheS*, *thrS*	9
GO:0006629	Lipid metabolic process	*dxs*, *glpQ*	2
GO:0006790	Sulfur compound metabolic process	*dxs*, *gor*	2
GO:0006810	Transport	*artI*, *copA*, *pcoC*, *ptsH*	4
GO:0006950	Response to stress	*sodB*, *ung*	2
GO:0007155	Cell adhesion	*fimA*	1
GO:0007165	Signal transduction	*artI*	1
GO:0009056	Catabolic process	*deoC*, *gcvH*, *gcvP*, *gcvT*, *glk*, *glpA*, *treC*, *uxaC*	8
GO:0009058	Biosynthetic process	*dxs*, *hisD*, *hns*, *ilvC*, *nadE*, *pdxY*, *rmlD*, *rnk*, *sul*, *udp*, *uvrD*	11
GO:0034641	Cellular nitrogen compound metabolic process	*cpdB*, *dxs*, *glk*, *gnd*, *gor*, *hisD*, *hns*, *nadE*, *pdxY*, *rmlB*, *rmlD*, *rnk*, *sul*, *talB*, *udp*	15
GO:0034655	Nucleobase-containing compound catabolic process	*cdd*, *cpdB*, *deoC*, *udp*	4
GO:0042592	Homeostatic process	*Gor*	1
GO:0044281	Small molecule metabolic process	*aspA*, *cdd*, *cpdB*, *deoC*, *dhaK*, *dhaL*, *dxs*, *fdhF*, *glk*, *glpA*, *gnd*, *nadE*, *pdxY*, *sul*, *talB*, *udp*, *uxaC*	17
GO:0051186	Cofactor metabolic process	*glk*, *gnd*, *nadE*, *pdxY*, *sul*, *talB*	6
GO:0051276	Chromosome organization	*uvrD*	1
GO:0051604	Protein maturation	*hscB*	1
GO:0055085	Transmembrane transport	*copA*	1
GO:0071554	Cell wall organization or biogenesis	*fimC*	1
**(B) Molecular functions**			
GO:0003677	DNA binding	*hns*, *rnk*, *uvrD*	3
GO:0003723	RNA binding	*gltX*, *pheS*, *thrS*	3
GO:0004386	Helicase activity	*uvrD*	1
GO:0004871	Signal transducer activity	*artI*	1
GO:0008168	Methyltransferase activity	*gcvT*	1
GO:0016301	Kinase activity	*dhaK*, *dhaL*, *glk*, *pdxY*, *ptsH*, *rnk*	6
GO:0016491	Oxidoreductase activity	*KPN_02441*, *fdhF*, *gcvP*, *glpA*, *gnd*, *gor*, *hisD*, *ilvC*, *nfnB*, *nfsA*, *rmlD*, *sodB*, *ydgJ*	13
GO:0016746	Transferring acyl groups	*Maa*	1
GO:0016757	Transferring glycosyl groups	*malP*, *udp*	2
GO:0016765	Transferring alkyl or aryl (other than methyl) groups	*Sul*	1
GO:0016791	Phosphatase activity	*aphA*, *gmhB*	2
GO:0016798	Acting on glycosyl bonds	*lacZ2*, *rihC*, *treC*, *ung*	4
GO:0016810	Acting on carbon–nitrogen (but not peptide) bonds	*Cdd*	1
GO:0016829	Lyase activity	*acnA*, *aspA*, *deoC*, *rmlB*, *yhbL*	5
GO:0016853	Isomerase activity	*ppiD*, *uxaC*	2
GO:0016874	Ligase activity	*gltX*, *nadE*, *pheS*, *thrS*	4
GO:0016887	ATPase activity	*copA*, *uvrD*	2
GO:0019899	Enzyme binding	*Rnk*	1
GO:0022857	Transmembrane transporter activity	*artI*, *copA*	2
GO:0030234	Enzyme regulator activity	*hscB*	1
**(B) Molecular functions**			
GO:0043167	Ion binding	*KPN_pKPN3p05899, aphA, cdd, copA, cpdB, dxs, fdhF, glk, glpA, gltX, gmhB, gor, hisD, ilvC, lacZ2, malP, nadE, pcoC, pdxY, pheS, sodB, sul, thrS, uvrD, yiiM*	25
**(C) Cellular component**			
GO:0005622	Intracellular	*artI*, *copA*, *lacZ2*, *ppiD*	1
GO:0005623	Cell	*Hns*	6
GO:0005737	Cytoplasm	*aphA*, *artI*, *fimA*, *fimC*, *gor*, *pcoC*	15
GO:0005886	Plasma membrane	*deoC*, *gcvH*, *glk*, *glpA*, *gltX*, *gmhB*, *maf*, *pheS*, *ptsH*, *talB*, *thrS*, *treC*, *udp*, *ung*, *uvrD*	2

### Protein Network Analysis

**FIGURE 1:**
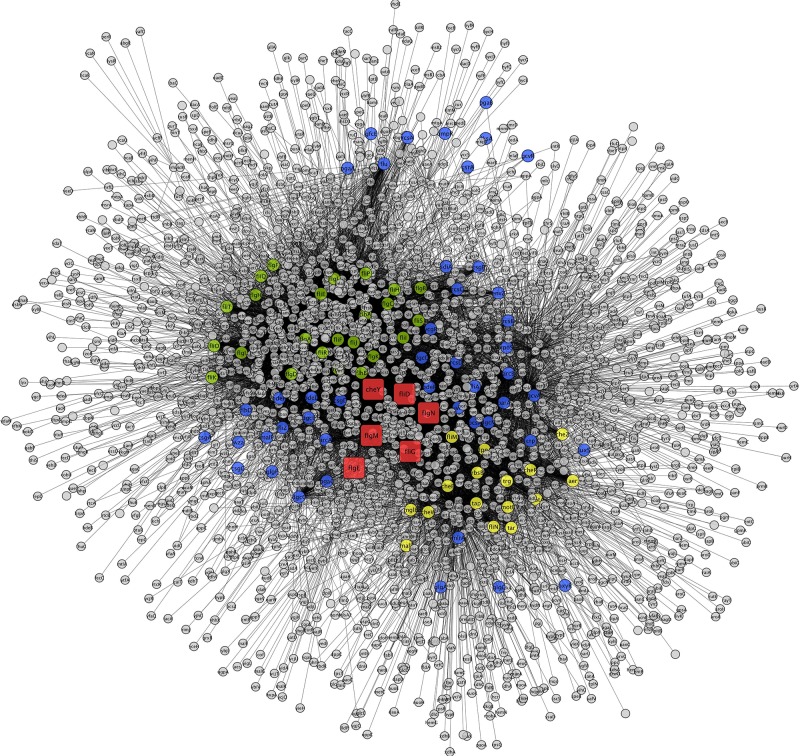
Interaction networks for down-regulated genes of *K. pneumoniae* using *E. coli* K12 substr. DH10B homologs. Interacting proteins were color coded by functions, biofilm formation (blue), chemotaxis proteins (yellow), and flagellar assembly (green). Square nodes in red color indicate down-regulated protein mapped on *E. coli*.

To construct the PPI network, the down-regulated proteins with motility-related functions were annotated using *E. coli* K12 strain DH10B homologs, as tabulated in Table 3. Among them, few proteins showed no hits in *E. coli*, and the rest of the proteins interacted with other proteins to make an interactome; this was visualized through Cytoscape (version 3.6.1) (Figure 1). Interacting proteins were color-coded by their functions: biofilm formation, blue; chemotaxis proteins, yellow; flagellar assembly, green. Square red nodes indicate down-regulated proteins.

**TABLE 3:** List of *Klebsiella pneumonia* sp. proteins mapped on *E. coli* K12 substr.DH10B.

***K. pneumoniae***	**Sequence**	***K. pneumoniae***	***E. coli* K12 substr.**	**Sequence**	***E. coli* K12 substr.**	**Identity (%)**
**IS22 protein entry**	**length**	**protein name**	**DH10B protein entry**	**length**	**DH10B gene name**	
W1AYD1	331	Flagellar motor switch protein FliG	P0ABZ1	331	fliG	99.698
W1AUQ1	206	Flagellar hook protein FlgE	P75937	402	flgE	91.304
W1AWJ3	97	Negative regulator of flagellin synthesis	P0AEM4	97	flgM	100.000
W1B018	468	Flagellar hook-associated protein 2	P24216	97	fliD	99.786
W1AP20	224	Chaperone FimC	No hit	–	–	–
W1ATC5	138	Flagellar biosynthesis protein FlgN	P43533	468	flgN	100.000
W1AT19	191	Flagellar basal body protein	P75937	138	flgE	95.812
W1BDF3	129	Chemotaxis regulator-transmits chemoreceptor signals to flagelllar motor components CheY	P0AE67	129	cheY	100.000
W1B0W2	533	Conjugal transfer protein traC	No hit	–	–	–
W1AZS9	447	Flagellin	No hit	–	–	–
A6T548	178	Putative fimbriae major subunit StbA	No hit			

## Discussion

The development of carbapenem-resistant *K. pneumoniae* has worsened the medical situation around the globe. The emergence of carbapenem resistance is usually due to the over-expression of carbapenemases and loss of porins. Recently, we reported a cluster of over-expressed proteins in carbapenem-resistant *K. pneumoniae* under meropenem pressure. These could be responsible for the drug resistance and belong to various categories such as the protein translational machinery complex, DNA/RNA modifying enzymes or proteins, proteins involved in carbapenem cleavage, modification, and transport, and energy metabolism- and intermediary metabolism-related proteins (Sharma et al., 2019a). Therefore, we suggested that they could be potential targets for the development of novel therapeutics against this resistance. Biofilm formation is also one of the mechanisms that leads to the development of drug resistance. In the present study, we have found a group flagellar, fimbriae, and pili proteins that are down-regulated under meropenem stress (sub-MIC). We hypothesize that the down-regulation of these proteins under meropenem stress makes the bacteria sessile or non-motile, leading to the emergence of a biofilm-like state that could contribute to carbapenem resistance in *K. pneumoniae* (NDM-4). Earlier microarray analysis of *K. pneumoniae* also reported the down-regulation of genes related to nitrogen metabolism, porin genes, and some membrane-associated proteins in association with the antibiotic resistance phenomenon (Doménech-Sánchez et al., 2006). The significance of the aforementioned pathways in antibiotic-evading mechanisms is also highlighted in several other reports (Yeung et al., 2011; Piek et al., 2014).

### A Hub of Flagellar, Fimbriae, and Pili Proteins Could Regulate Resistance

A group of flagellar, fimbriae, and pili proteins involved in the formation of the flagellar machinery complex and the regulation of motility processes were found to be down-regulated in meropenem-induced carbapenem-resistant *K. pneumoniae* clinical strains. These proteins are flagellar motor switch protein FliG, flagellar hook protein FlgE, negative regulator of flagellin synthesis FlgM, putative fimbriae major subunit StbA, flagellar hook-associated protein 2, chaperone FimC protein, flagellar biosynthesis protein FlgN, flagellar basal body protein FlgF, conjugal transfer protein TraC, fimbrial subunit type 1, chemotaxis regulator-transmits chemoreceptor signals to flagellar motor components CheY, and flagellin.

The observed expression down-regulation of flagella-, fimbriae-, and pili-related proteins provides clues to a novel mechanism of drug resistance. Flagellin, flagellar biosynthesis protein FlgN, and FlgM, respectively, cumulatively maintain equilibrium in the biosynthesis of flagella. Flagellin protein is part of the structural component of flagella, and biosynthesis of flagella is favored by flagellar biosynthesis protein FlgN (Bennett et al., 2001). FlgM is a negative regulator that switches off flagellar transport. FlgM can be exported from the cell via a flagellum, and the transport occurs only after the completion of hook formation (Hughes et al., 1993). This unique regulatory mechanism further postpones flagellin synthesis in the cell. Cumulatively, the expression of these proteins is involved in the flagella formation, regulation, and motility of the bacteria. Therefore, we suggest that, under meropenem pressure, down-regulation of these proteins might make the bacteria sessile, which is the first step in biofilm-formation. Therefore, we assume that down-regulation of these proteins could create a biofilm-like state, which ultimately leads to the drug resistance.

Flagella are composed of three different parts: a filament (helical and long), hook (a curved and short structure), and basal body (a complex structure with a central rod and a series of rings). Flagellar motor switch protein (FliG), flagellar hook protein FlgE, flagellar hook-associated protein 2, and flagellar basal body protein FlgF together make up part of the flagellar motor switch complex (FliG, FliN, and FliM), which is involved in bacterial motility, after receipt and transduction of the signal by chemotaxis (Djordjevic and Stock, 1998). This is a complex apparatus that senses the signal from the chemotaxis sensory signaling system and is transduced into motility. Chemotaxis response regulator CheY transmits chemoreceptor signals to flagellar motor components and is believed to be the “on” switch that directly induces tumbles in the swimming pattern (Robinson et al., 2000). Physical interactions of CheY and switch proteins have not been reported. Chemotactic stimuli change the association of the CheY signal protein with the distal FliM_NC_FliN C ring (Dyer et al., 2009; Sarkar et al., 2010). In the present study, down-regulation of the chemotaxis response regulator (CheY) subsequently down-regulates signal transmission to the flagellar motor components, which may act as an “off” switch and make the bacteria sessile or non-motile. Further, it might lead to a biofilm-like state and could contribute to the drug resistance.

**FIGURE 2:**
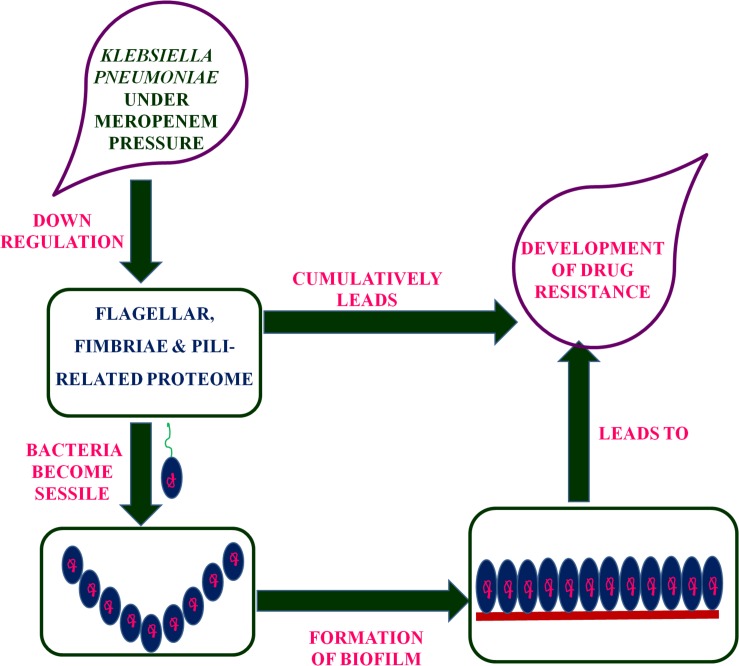
Proposed model based on the flagellar-, fimbriae-, and pili-related proteome suggested the mechanism of carbapenem-resistance in *K. pneumoniae* (NDM-4) clinical isolates.

Putative fimbriae major subunit StbA, Fimbrial subunit type 1, conjugal transfer protein TraC, and chaperone FimC protein are involved in pillus organization, fimbriae formation, and their associated assemblies (Johnson and Clegg, 2010). FimC protein acts as a periplasmic pilin chaperone that not only protects the bacteria under stress through chaperone-mediated folding but is also involved in pillus formation (Klemm, 1992). In the periplasm, the FimC chaperone binds to the major and minor structural components and protects them from degradation. Conjugal transfer protein TraC, encoded by a gene, *traC*, presents on plasmid and is involved in the conjugation process as well as pili formation (Winans and Walker, 1985; Kado, 1994), leading to the transfer of drug-resistant plasmid to bacteria. These down-regulated proteins (flagellar, fimbriae, and pili proteins) form a hub of proteins in the PPI network, which indicates their important role in flagellar, fimbriae, and pili assemblies, signaling through chemotaxis proteins, and biofilm formation (Figure 1). In this study, the down-regulation of fimbriae-, pili-, and conjugative-related proteins leads to the creation of the biofilm-like state, which may contribute to drug resistance. On the basis of the flagella-, fimbriae-, and pili-related proteome, we propose a model (Figure 2) that suggests the potential path or mechanism of carbapenem resistance in *K. pneumoniae* (NDM-4) clinical isolates. Overall this group of down-regulated proteins and their interactive protein partners cumulatively make a hub that leads to the formation of a biofilm-like scenario and could contribute to meropenem resistance.

## Conclusion

In brief, the present study focused on the down-regulated proteome of carbapenem-resistant *K. pneumoniae* clinical isolate (NDM-4) under meropenem pressure through proteomics and systems biology approaches. A group of down-regulated proteins was identified that belongs to the flagellar, fimbriae, and pili proteins. Therefore, we suggest that these proteins and their interactive protein partners cumulatively lead to the bacteria becoming sessile, which further creates a biofilm-like state and could contribute to the survival of bacteria under meropenem pressure, which might reveal a novel mechanism of drug resistance. Further research on these motility-related protein targets and their pathways may lead to the development of novel therapeutics against the worsening situation of drug resistance.

## Data Availability Statement

The raw data supporting the conclusions of this article will be made available by the authors, without undue reservation, to any qualified researcher.

## Author Contributions

DS designed the concept, and experimented and wrote the manuscript. AG and MK carried out the systems biology analysis. FR provided support in the LC–MS experiments and analysis. AK designed and guided the study and finalized the manuscript. All authors approved the final manuscript.

## Conflict of Interest

FR was employed by SCIEX Pvt. Ltd. The remaining authors declare that the research was conducted in the absence of any commercial or financial relationships that could be construed as a potential conflict of interest.

## Footnotes

1 http://www.geneontology.org/

2 http://www.string-db.org/

## Abbreviations

CLSI: Clinical and Laboratory Standards Institute
ESBLs: extended spectrum beta-lactamases
LB: Luria–Bertani
MIC: minimum inhibitory concentration
STRING: Search Tool for the Retrieval of Interacting Genes/Proteins.
