# Supplementary material for: Down-Regulation of Flagellar, Fimbriae, and Pili Proteins in Carbapenem-Resistant Klebsiella pneumoniae (NDM-4) Clinical Isolates: A Novel Linkage to Drug Resistance
Source: Front Microbiol. 2019 Dec 17;10:2865. doi: 10.3389/fmicb.2019.02865 (PMC6928051; doi:10.3389/fmicb.2019.02865)
Supplement: Supplementary file 1 [file Table_1.doc]

**Supplementary Table S1:** Details of the down-regulated proteome (less than 0.42 folds) under meropenem stress in *Klebsiella pneumonia* clinical isolates (NDM-4).

| S.No. | **Protein name** | **Log fold change vs. P-value** | **Accession number** |
| --- | --- | --- | --- |
| 1 | Flagellar motor switch protein FliG | 0.42 | W1AYD1 |
| 2 | Uncharacterized protein conserved in bacteria | 0.41 | A6T5B2 |
| 3 | Putative translation initiation inhibitor YoaB | 0.41 | A6TAY1 |
| 4 | L-arabinose 1-dehydrogenase | 0.40 | W1B7V1 |
| 5 | YhdH, a putative quinoneoxidoreductase | 0.40 | W1ARQ8 |
| 6 | Maf-like protein | 0.39 | A6TER4 |
| 7 | Glycerophosphoryldiesterphosphodiesterase, periplasmic | 0.39 | A6TBU1 |
| 8 | NMN phosphatase Class B acid phosphatase | 0.39 | A6TGW3 |
| 9 | Phosphatase NudJ | 0.38 | A6T7K4 |
| 10 | Aconitatehydratase | 0.37 | A6T4R3 |
| 12 | Phosphoenolpyruvate-dihydroxyacetonephosphotransferase, ADP-binding subunit DhaL | 0.36 | A6TEA7 |
| 13 | Glutamate decarboxylase | 0.36 | W1AXH1 |
| 14 | Non-specific ribonucleoside hydrolase RihC | 0.35 | A6T4G4 |
| 15 | Cytidinedeaminase | 0.34 | A6TBN1 |
| 16 | 2',3'-cyclic-nucleotide 2'-phosphodiesterase | 0.32 | A6THC4 |
| 17 | Flagellar hook protein FlgE | 0.32 | W1AUQ1 |
| 18 | Uracil-DNA glycosylase | 0.32 | A6TCJ2 |
| 19 | Trehalose-6-phosphate hydrolase | 0.31 | A6THG8 |
| 20 | Glycine cleavage system H protein | 0.31 | A6TDR6 |
| 21 | Aminomethyltransferase | 0.30 | A6TDR7 |
| 22 | Glucokinase, ROK family | 0.30 | A6TC33 |
| 23 | Methyl-accepting chemotaxis protein I (Serine chemoreceptor protein) | 0.30 | W1APM1 |
| 24 | Uridinephosphorylase | 0.29 | A6TGL1 |
| 25 | dTDP-4-dehydrorhamnose reductase | 0.29 | A6TBD6 |
| 26 | 1-deoxy-D-xylulose-5-phosphate synthase | 0.29 | A6T5F3 |
| 27 | Formate dehydrogenase H @ selenocysteine-containing | 0.29 | A6TA97 |
| 28 | Dihydropteroate synthase | 0.27 | A6TIZ0 |
| 29 | Formatehydrogenlyase subunit 2 | 0.25 | A6TCZ1 |
| 30 | Negative regulator of flagellin synthesis FlgM | 0.23 | W1AWJ3 |
| 31 | Glycine dehydrogenase (Aminomethyl-transferring) | 0.23 | A6TDR5 |
| 32 | Putative fimbriae major subunit StbA | 0.23 | A6T548 |
| 33 | Flagellar hook-associated protein 2 | 0.22 | W1B018 |
| 34 | Phage shock protein E | 0.22 | W1B7B7 |
| 35 | Uncharacterized protein | 0.22 | A6TH03 |
| 36 | Transaldolase, tal | 0.19 | A6T4E8 |
| 37 | dTDP-glucose 4,6-dehydratase | 0.18 | A6TBD8 |
| 38 | Chaperone protein hchA | 0.18 | W1B0V3 |
| 39 | Arginine ABC transporter, periplasmic arginine-binding protein ArtI | 0.18 | A6T6W1 |
| 40 | t-RNA Sulfurtransferase | 0.17 | A6T5F6 |
| 41 | Uncharacterized protein | 0.17 | A1XPK1 |
| 42 | UPF0757 protein YmgG | 0.17 | W1BBU7 |
| 43 | Oxygen-insensitive NADPH nitroreductase | 0.16 | A6T6U7 |
| 44 | Protein phosphatase CheZ | 0.15 | W1BFA5 |
| 45 | Chaperone FimC | 0.15 | W1BBF2 |
| 46 | DNA-binding protein | 0.14 | A6TAK8 |
| 47 | Uncharacterized protein, BN49_4751 | 0.13 | W9BQD8 |
| 48 | Uncharacterized protein | 0.13 | A6T8V8 |
| 49 | ATP-dependent Clp protease ATP-binding subunit | 0.13 | A6T5I1 |
| 50 | Peptidylprolylisomerase | 0.12 | A6T5I4 |
| 51 | Glucose-1-phosphatase | 0.12 | A6T791 |
| 52 | Oxygen-insensitive NAD(P)H nitroreductase / Dihydropteridinereductase | 0.12 | A6T5Y2 |
| 53 | D,D-heptose 1,7-bisphosphate phosphatase | 0.11 | A6T503 |
| 54 | Uncharacterized protein | 0.11 | A6T6F0 |
| 55 | Uncharacterized protein | 0.10 | W1B791 |
| 56 | UPF0098 protein ybhB | 0.09 | A6T6L3 |
| 57 | Co-chaperone protein HscB | 0.09 | A6TCE8 |
| 58 | Flagellar biosynthesis protein FlgN | 0.09 | W1ATC5 |
| 59 | Flagellar basal body protein | 0.08 | W1AT19 |
| 60 | Uncharacterized protein | 0.08 | A0A231WIF6 |
| 61 | Uncharacterized protein | 0.07 | E0W6V6 |
| 62 | Conjugal transfer protein traC | 0.06 | W1B0W2 |
| 63 | Copper resistance protein C | 0.06 | A6TI95 |
| 64 | Fimbrial subunit type 1 | 0.05 | W1B9X4 |
| 65 | Ketol-acid reductoisomerase | 0.05 | A6TGG1 |
| 66 | Small HspC2 heat shock protein | 0.05 | A6TI56 |
| 67 | Chemotaxis regulator-transmits chemoreceptor signals to flagellar motor components CheY | 0.04 | W1BDF3 |
| 68 | DUF305 domain-containing protein | 0.04 | A0A343YQ07 |
| 69 | Flagellin | 0.01 | W1AZS9 |

**Supplementary Table S2:** List of down-regulated genes mapped on *Klebsiella pneumonia* subsp*.* pneumonia (strain ATCC 700721/MGH 78578). All *in-silico* functional annotation work was carried out with these proteins*.*

| **S. No.** | **Protein name** | **Accession**  **Number** | **Gene name** | **Log fold change vs. P-value** |
| --- | --- | --- | --- | --- |
|  | Transaldolase (EC 2.2.1.2) | A6T4E8 | talB | 0.19 |
|  | Non-specific ribonucleoside hydrolase RihC (EC 3.2.-.-) (Purine/pyrimidine ribonucleoside hydrolase) | A6T4G4 | rihC | 0.35 |
|  | D, D-heptose 1,7-bisphosphate phosphatase (EC 3.1.3.-) | A6T503 | gmhB | 0.11 |
|  | 1-deoxy-D-xylulose-5-phosphate synthase (EC 2.2.1.7) (1-deoxyxylulose-5-phosphate synthase) (DXP synthase) (DXPS) | A6T5F3 | dxs | 0.29 |
|  | Peptidylprolylisomerase (EC 5.2.1.8) | A6T5I4 | ppiD | 0.12 |
|  | Maltose O-acetyltransferase | A6T5M1 | maa | 0.44 |
|  | Cu(I)-translocating P-type ATPase | A6T5P4 | copA | 0.45 |
|  | Dihydropteridinereductase/oxygen-insensitive NAD(P)H nitroreductase | A6T5Y2 | nfnB | 0.12 |
|  | Regulator of nucleoside diphosphate kinase | A6T682 | rnk | 0.50 |
|  | Glutamate/aspartate periplasmic binding protein | A6T6B0 | gltI | 0.47 |
|  | Conserved protein, phosphatidylethanolamine-binding domain | A6T6L3 | ybhB | 0.09 |
|  | Oxygen insensitive NADPH nitroreductase (Nitrofuranreductase I activity B) | A6T6U7 | nfsA | 0.16 |
|  | Arginine 3rd transport system periplasmic binding protein | A6T6W1 | artI | 0.18 |
|  | Glucose-1-phosphatase | A6T791 | agp | 0.12 |
|  | Phosphatase NudJ (EC 3.6.1.-) | A6T7K4 | ymfB | 0.38 |
|  | Sulfurtransferase | A6T7S5 | ynjE | 0.17 |
|  | NH(3)-dependent NAD(+) synthetase (EC 6.3.1.5) | A6T7U0 | nadE | 0.47 |
|  | Aconitatehydratase (Aconitase) (EC 4.2.1.3) | A6T7Y4 | acnA | 0.37 |
|  | Hypothetical oxidoreductase | A6T9W9 | ydgJ | 0.40 |
|  | Pyridoxal kinase PdxY (PL kinase) (EC 2.7.1.35) | A6T9Y4 | pdxY | 0.49 |
|  | Superoxide dismutase (EC 1.15.1.1) | A6TA04 | sodB | 0.49 |
|  | Phenylalanine--tRNA ligase alpha subunit (EC 6.1.1.20) (Phenylalanyl-tRNAsynthetase alpha subunit) (PheRS) | A6TAI3 | pheS | 0.42 |
|  | Threonine--tRNA ligase (EC 6.1.1.3) (Threonyl-tRNAsynthetase) (ThrRS) | A6TAI7 | thrS | 0.49 |
|  | DNA-binding protein | A6TAK8 | hns | 0.14 |
|  | Uncharacterized protein | A6TAY1 | yoaB | 0.41 |
|  | Putative dehydrogenase | A6TB90 | KPN_02441 | 0.40 |
|  | Histidinol dehydrogenase (HDH) (EC 1.1.1.23) | A6TBC3 | hisD | 0.50 |
|  | dTDP-4-dehydrorhamnose reductase (EC 1.1.1.133) | A6TBD6 | rmlD | 0.29 |
|  | 6-phosphogluconate dehydrogenase, decarboxylating (EC 1.1.1.44) | A6TBE6 | gnd | 0.02 |
|  | Cytidinedeaminase (EC 3.5.4.5) (Cytidineaminohydrolase) (CDA) | A6TBN1 | cdd | 0.34 |
|  | Glycerophosphodiesterphosphodiesterase, periplasmic | A6TBU1 | glpQ | 0.39 |
|  | Glycerol-3-phosphate dehydrogenase (EC 1.1.5.3) | A6TBU3 | glpA | 0.46 |
|  | Glucokinase (EC 2.7.1.2) (Glucose kinase) | A6TC33 | glk | 0.30 |
|  | Glutamate--tRNA ligase (EC 6.1.1.17) (Glutamyl-tRNAsynthetase) (GluRS) | A6TC43 | gltX | 0.44 |
|  | PTS family Hpr protein, phosphohistidinoprotein-hexose phosphotransferase | A6TC51 | ptsH | 0.45 |
|  | Co-chaperone protein HscB (Hsc20) | A6TCE8 | hscB | 0.09 |
|  | Uracil-DNA glycosylase (UDG) (EC 3.2.2.27) | A6TCJ2 | ung | 0.32 |
|  | Hydrogenase-3, Fe-S subunit (Part of FHL complex) | A6TCZ1 | hycB | 0.25 |
|  | Major type 1 subunit fimbrin (Pilin) | A6TDL4 | fimA | 0.05 |
|  | Periplasmic chaperone | A6TDL6 | fimC | 0.17 |
|  | Glycine dehydrogenase (decarboxylating) (EC 1.4.4.2) (Glycine cleavage system P-protein) (Glycine decarboxylase) (Glycine dehydrogenase (aminomethyl-transferring)) | A6TDR5 | gcvP | 0.43 |
|  | Glycine cleavage system H protein | A6TDR6 | gcvH | 0.31 |
|  | Aminomethyltransferase (EC 2.1.2.10) (Glycine cleavage system T protein) | A6TDR7 | gcvT | 0.30 |
|  | Putative dihydroxyacetone kinase, N-terminal domain | A6TEA6 | dhaK | 0.45 |
|  | Putative dihydroxyacetone kinase, C-terminal domain | A6TEA7 | dhaL | 0.36 |
|  | Uronateisomerase (EC 5.3.1.12) (Glucuronateisomerase) (Uronicisomerase) | A6TED1 | uxaC | 0.49 |
|  | Glyoxalase | A6TEM7 | yhbL | 0.43 |
|  | Uncharacterized protein | A6TEQ5 | yhcN | 0.46 |
|  | Maf-like protein KPN_03656 | A6TER4 | maf | 0.39 |
|  | Alpha-1,4glucanphosphorylase (EC 2.4.1.1) | A6TF40 | malP | 0.48 |
|  | Glutathione reductase | A6TFB9 | gor | 0.44 |
|  | Uncharacterized protein | A6TGB3 | yiiM | 0.41 |
|  | Ketol-acid reductoisomerase (NADP(+)) (KARI) (EC 1.1.1.86) (Acetohydroxy-acid isomeroreductase) (AHIR) (Alpha-keto-beta-hydroxylacylreductoisomerase) (Ketol-acid reductoisomerase type 2) (Ketol-acid reductoisomerase type II) | A6TGG1 | ilvC | 0.32 |
|  | dTDP-glucose 4,6-dehydratase (EC 4.2.1.46) | A6TGH6 | rmlB | 0.43 |
|  | DNA helicase (EC 3.6.4.12) | A6TGJ6 | uvrD | 0.49 |
|  | Uridinephosphorylase (EC 2.4.2.3) | A6TGL1 | udp | 0.29 |
|  | Class B acid phosphatase (EC 3.1.3.2) | A6TGW3 | aphA | 0.39 |
|  | Uncharacterized protein | A6TH03 | fdhF | 0.29 |
|  | Aspartate ammonia-lyase (Aspartase) (EC 4.3.1.1) | A6TH49 | aspA | 0.43 |
|  | 2',3'-cyclic nucleotide 2'-phosphodiesterase/3'-nucleotidase bifunctionalperiplasmic protein | A6THC4 | cpdB | 0.32 |
|  | Trehalase 6-P hydrolase | A6THG8 | treC | 0.31 |
|  | Deoxyribose-phosphate aldolase (DERA) (EC 4.1.2.4) (2-deoxy-D-ribose 5-phosphate aldolase) (Phosphodeoxyriboaldolase) (Deoxyriboaldolase) | A6THZ6 | deoC | 0.49 |
| 63. | Beta-galactosidase 2 (Beta-gal 2) (EC 3.2.1.23) (Lactase 2) | A6TI29 | lacZ2 | 0.50 |
| 64. | Molecular chaperone (Small heat shock protein) | A6TI56 | KPN_pKPN3p05898 | 0.10 |
| 65. | ATPase with chaperone activity, ATP-binding subunit | A6TI57 | KPN_pKPN3p05899 | 0.13 |
| 66. | Copper resistant protein PcoC | A6TI95 | pcoC | 0.06 |
| 67. | Dihydropteroate synthase (DHPS) (EC 2.5.1.15) (Dihydropteroatepyrophosphorylase) | A6TIZ0 | sul | 0.27 |
